# Improved Cognitive Promotion through Accelerated Magnetic Stimulation

**DOI:** 10.1523/ENEURO.0392-20.2020

**Published:** 2021-02-01

**Authors:** Xingqi Wu, Lu Wang, Zhi Geng, Ling Wei, Yibing Yan, Chengjuan Xie, Xingui Chen, Gong-Jun Ji, Yanghua Tian, Kai Wang

**Affiliations:** 1 Department of Neurology, the First Affiliated Hospital of Anhui Medical University, Hefei 230022, China; 2 Anhui Province Key Laboratory of Cognition and Neuropsychiatric Disorders, Hefei 230022, China; 3 Collaborative Innovation Center of Neuropsychiatric Disorders and Mental Health, Hefei 230022, China; 4 Department of Neurology, Second People’s Hospital of Hefei City, The Hefei Affiliated Hospital of Anhui Medical University, Hefei 230022, China; 5 Department of Medical Psychology, Chaohu Clinical Medical College, Anhui Medical University, Hefei 230022, China

**Keywords:** cognition enhancement, executive function, intermittent theta-burst stimulation, non-invasive brain stimulation, repetitive transcranial magnetic stimulation, working memory

## Abstract

Noninvasive brain stimulation to enhance cognition is an area of increasing research interest. Theta burst stimulation (TBS) is a novel accelerated form of stimulation, which more closely mimics the brain’s natural firing patterns and may have greater effects on cognitive performance. We report here the comparative assessment of the effect of conventional high-frequency repetitive transcranial magnetic stimulation (HF-rTMS) protocols and TBS protocols on cognition enhancement in healthy controls. Sixty healthy adults (34 males and 26 females) were randomized and counterbalanced and assigned to HF-rTMS (*n* = 20), TBS (*n* = 20), or sham (*n* = 20) groups. The promotion effects of different parameters of prefrontal stimulation on working memory and executive function were compared, as assessed by performance in N-back tasks and the Wisconsin Card Sorting Test (WCST). Both HF-rTMS and intermittent TBS (iTBS) groups displayed a significant improvement in N-back tasks, with an effect size of 0.79 and 1.50, respectively. Furthermore, the iTBS group displayed a significant improvement in the WCST, with an effect size of 0.84. The iTBS group demonstrated higher effect sizes than the HF-rTMS group (*t *=* *2.68, *p *=* *0.011), with an effect size of 0.85. However, no improvement in other tasks was observed (*p *>* *0.05). Intermittent TBS has a stronger cognitive promoting effect than conventional rTMS. In summary, our findings provide direct evidence that iTBS may be a superior protocol for cognitive promotion.

## Significance Statement

This study presents a randomized, placebo-controlled, parallel, double-blinded comparison of the effects of 20-Hz repetitive transcranial magnetic stimulation (rTMS) and intermittent theta burst stimulation (iTBS) applied over the left dorsolateral prefrontal cortex (DLPFC), on working memory and executive functions. Significant improvements in working memory performance were observed following active 20-Hz rTMS and iTBS, the effect size of which were 0.71 and 1.50, respectively. The improvement of executive function (cognitive flexibility) performance was observed following iTBS, but not in 20-Hz rTMS. This study demonstrated that iTBS has a stronger cognitive promoting effect than 20-Hz rTMS and provided direct evidence that iTBS may be a better protocol for cognitive promotion.

## Introduction

Cognitive deficits can be caused by numerous neuropsychiatric disorders, such as Alzheimer’s disease, Parkinson’s disease, major depression disease (MDD) and schizophrenia (Iimori et al., 2019; Chou et al., 2020). The impairment of daily living and social functioning resulting from cognitive dysfunction imposes a severe financial burden on families and society (Feigin et al., 2017). Therefore, finding suitable cognitive promotion methods is an important topic for neuroscience research (Chou et al., 2020; Momi et al., 2020).

Repetitive transcranial magnetic stimulation (rTMS) may be a promising alternative that can modify neural activity non-invasively (Koch et al., 2018; Noda et al., 2018; Chou et al., 2020). Over the last several decades, research on the use of rTMS for the enhancement of cognition has dramatically increased. The neural effects of rTMS depend on the frequency of stimulation. When rTMS is pulsed at a low frequency (∼1 Hz), the long-term depression (LTD) of synaptic connections and cortical excitability generally decrease, while higher frequency rTMS (usually between 5 and 20 Hz) can lead to the long-term potentiation (LTP) of synaptic connections and increase cortical excitability (Parkin et al., 2015). Various studies have demonstrated that high-frequency rTMS (HF-rTMS) stimulation of the left dorsolateral prefrontal cortex (DLPFC) improves cognitive functions in healthy adults and neuropsychiatric patients, such as working memory, attention, and executive control (Curtin et al., 2019; Iimori et al., 2019). Furthermore, Guse and colleagues found that rTMS at 20 Hz, applied over the left DLPFC, is more likely to cause significant cognitive improvement than 10- and 15-Hz rTMS (Guse et al., 2010).

The fundamental basis for brain function is brain oscillations in various frequency bands (Henry et al., 2014). Several studies provide evidence supporting the idea that modulating oscillations via frequency-specific entrainment can alter cognition in healthy individuals (Roberts et al., 2018). Furthermore, rTMS has been shown to improve cognitive function through the same mechanism (Thut et al., 2011a,b). Particularly, several studies suggest that focal rTMS may promote entrainment and synchronization of human brain oscillations, which may boost cognitive correlates (Thut et al., 2011a). According to Hoy and collogues, neural synchrony refers to the coordinated firing of connected brain regions and is considered essential for the integration of neural networks and cognitive performance (Hoy et al., 2016). In other words, rTMS can be an effective oscillatory entrainment approach with the potential to improve specific cognitive functions like attention and perception (Thut et al., 2011b).

The intermittent theta burst transcranial magnetic stimulation paradigm (iTBS) is an accelerated form of HF-rTMS, which is thought to more robustly alter cortical excitability than the standard, non-accelerated approach, because of the mimicking of the brain’s natural firing patterns (Paulus, 2005; Suppa et al., 2016). The superior effects of iTBS, compared with that of HF-rTMS, are thought to be because of the more “naturalistic” pattern of stimulation provided with iTBS, which is based on *in vivo* patterns of pyramidal neuronal firing associated with LTP induction (Oberman et al., 2011).

Theta oscillations are associated with many cognitive processes, such as working memory and episodic memory (Thut and Miniussi, 2009; Roberts et al., 2018; Griffiths et al., 2019). Electrophysiological recordings in rodents have established the role of the theta band in the hippocampal-PFC in spatial working memory (Gordon, 2011). Specifically, theta-γ coupling coherence between both structures is elevated during increased working memory demands; this is defined as the increasing phase-locking of prefrontal spike-timing to the hippocampal theta rhythm (Jones and Wilson, 2005). Furthermore, human imaging studies have revealed increased theta-γ oscillation coupling between the hippocampus and the DLPFC during working memory (Axmacher et al., 2008; Bähner et al., 2015). Thus, specific theta rhythmic stimulation may ameliorate the degree of coherence in spiking neurons between the hippocampal formation and the PFC, which facilitates the induction of the working memory process (or hinders that process). Noda and colleagues previously reported that HF-rTMS on the left DLPFC induced a significant increase of theta-γ coupling between DLPFC and the hippocampus and within DLPFC itself (Noda et al., 2017, 2018). Furthermore, this increase in theta-γ phase-to-amplitude coupling is significantly associated with the improved accuracy rate (ACC) of executive function, as assessed by the Wisconsin Card Sorting Test (WCST) following rTMS treatment in MDD (Noda et al., 2017, 2018). Here, we hypothesize that iTBS on DLPFC may induce more improvement than conventional HF-rTMS in working memory and executive functions.

## Materials and Methods

### Participants

Seventy-one healthy adults (39 males and 32 females) were initially recruited from Anhui Medical University. Eleven participants were disqualified because of personal reasons or a refusal to complete the scanning. Ultimately, 60 adults (34 males and 26 females) completed the experiments. Criteria for exclusion included a history of any psychiatric or neurologic illness (*n* = 1), seizure (*n* = 2), any serious medical conditions, current pregnancy, or refusal to participate in all the tests and MRI scans (*n* = 11, refused to undergo the neuropsychological assessment, MRI scans, or TMS stimulation). All participants were TMS naive. This study protocol was reviewed and approved by the Medical Ethics Committee of Anhui Medical University. All participants provided informed written consent.

### Experimental design

**Figure 1.**
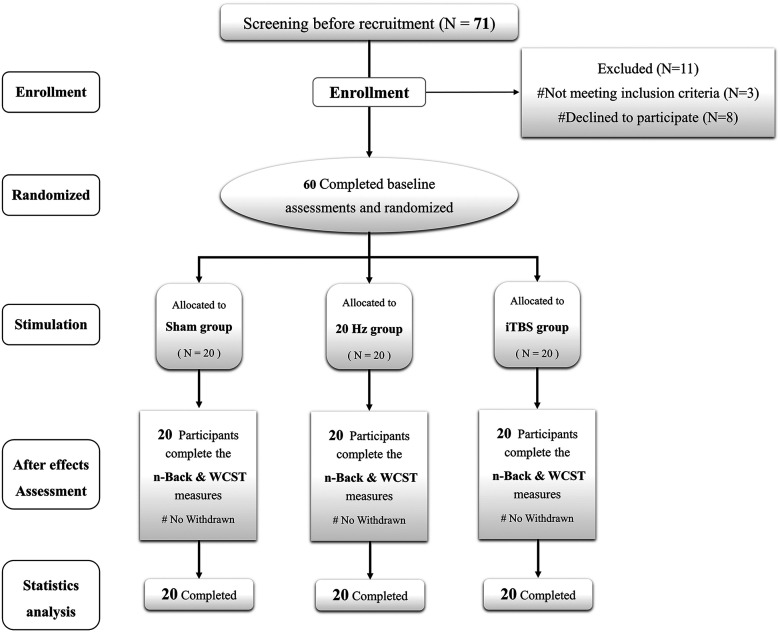
The flowchart and diagrammatic representation of the timeline for the study. Of the 71 healthy participants who completed screening, 60 subjects were assigned to HF-rTMS (*n* = 20), iTBS (*n* = 20), or sham (*n* = 20) groups. Three participates do not meet the inclusion criteria, eight participants decline take part in the study.

This study used a randomized, sham-controlled, parallel, double-blinded design (Fig. 1). Before the experiment, participants were randomized and counterbalanced by using a random number table, into the following groups: HF-rTMS (*n* = 20), iTBS (*n* = 20), or sham (*n* = 20). On the first experiment day, structure images, a battery of neuropsychology assessments, and the baseline performance of N-back and WCST were acquired. On the second experiment day, the resting motor threshold (RMT) was determined, and TMS with different parameters was subsequently applied to the left DLPFC (described below, Study interventions: image-navigated rTMS). Participants then undertook the N-back tasks and WCST immediately following stimulation; there was no delay after stimulation, and the N-back tasks were undertaken first. To ensure the double-blinded design, participants were instructed not to discuss their treatment allocation with the staff or other participants.

### Neuropsychology assessments

A battery of neuropsychology assessments was performed at the first visit to evaluate global cognition, emotion, memory, attention, executive functions, and fluency for frontal lobe function of all participants. The battery of neuropsychology assessments included the Montreal Cognitive Assessment (MoCA, Beijing-version), Hamilton Anxiety Rating Scale-14 items (HAMA), Hamilton Depression Rating Scale-17 items (HAMD), Chinese version of the Auditory Verbal Learning Test (AVLT), Digital Span Test (forward/backward, DST-F/B), Stroop Color Word Test (SCWT), Color Trail Test A/B (CTT-A/B), and Verbal Fluency Test (VFT–letter and semantic, VFT-L/S).

### Study interventions: image-navigated rTMS

TBS was performed using a Magstim Rapid^2^ transcranial magnetic stimulator (Magstim Company) with a 70-mm air-cooled figure-of-eight coil. The RMT was estimated for each subject to set the individualized stimulation strength before the TMS. RMT was defined as the lowest intensity that evoked a small response (>50 mV) in at least five of 10 consecutive trials, as measured from the right first dorsal interosseous muscle by a handheld 70-mm figure-of-eight coil (Huang et al., 2005).

The HF-rTMS parameters were adopted as follows: delivered at 110% of the participant’s RMT via 45 trains of 2-s 20-Hz rTMS (i.e., 40 pulses per train), each of which was followed by intertrain pauses of 28 s, for 22.5 min and a total of 1800 pulses (Eldaief et al., 2011). The iTBS parameters were adopted as follows: 70% of RMT stimulus was delivered in the form of bursts at a frequency of 5 Hz, with each burst consisting of three stimuli delivered at a frequency of 50 Hz, a total of 10 bursts, and a total of 600 stimuli per session (Barr et al., 2009). The cumulative effect of rTMS is a temporary modulation of cortical excitability in the targeted cortical region and its associated networks, which affects post-rTMS task performance as compared with that at pre-rTMS baseline (Huang et al., 2007). To achieve a better cumulative aftereffect, three typical iTBS were delivered three times at 15-min intervals with a total of 1800 stimulus (Shirota et al., 2017). The sham stimulus was delivered with a placebo coil (MagStim Company) that produced a similar sound and sensation on the scalp as the real coil but did not induce a current in the cortex. The protocol of sham stimulus was the same as for iTBS.

The TMS stimulation was delivered to the left DLPFC at Montreal Neurologic Institute coordinates [–38 44 26] (Mir-Moghtadaei et al., 2015). To accurately target the coil placement, all stimulations were guided by the participant’s anatomic image (1 × 1 × 1 mm^3^) and a frameless neuro-navigation system (Brainsight; Rogue Research). The coil was placed with the aid of Visor 2.0 (ANT Neuro) and held tangentially to the skull with the handle pointing posterolaterally. The middle point of the figure-of-eight MagStim coil was placed directly over the region of interest.

The adverse effects were recorded by self-report and operator monitoring, including painful scalp sensations, eyelid twitch, tinnitus, epilepsy, and epileptic seizures. When questioned at the end of the study, none of the participants were certain of their group assignment.

### Outcome measures

#### Working memory task: visual-spatial N-back

Participants performed the 20-min visual-spatial N-back task at baseline and immediately following stimulation. The visual-spatial N-back task was administered using E-Prime 1.0 (Psychology Software Tools). The N-back task consisted of 0-back, 1-back, 2-back, and 3-back tasks, each task being performed three times and the 12 parts performed pseudo-randomly. Each task contained 60 targets. In the 0-back, 1-back, 2-back, and 3-back tasks, a sequence of 60 stimuli was presented, each consisting of the same visual object (color blocks) randomly presented in one of four possible locations on the black screen in a consecutive manner. The locations were arranged as up, down, left, right, and in the center of the screen, and each stimulus was presented for 0.5 s with an interstimulus interval of 1.5 s. The participants were asked to indicate the position of stimulus in the current trial, or one, two, or three steps earlier, by pressing a button. Working memory performance was assessed using the AAC (%) and reaction time (RT; ms; Owen et al., 2005; Bagherzadeh et al., 2016).

#### WCST

The WCST was performed at baseline and at the end of stimulation. The WCST was considered a measure of executive functions, which is extensively used in neuropsychological testing. The task provides information on several aspects of executive function beyond basic indices as task success [total responses (TR), total correct (TC)] or task failure (total errors; TE). Important indices of performance on the task include the perseverative error responses percentage (PERP; i.e., the percentage of failures to shift cognitive sets in response to negative feedback), which reflects the cognitive flexibility (set-shifting) of subjects (Kopp et al., 2021). We evaluated the executive functions by using indices of the WCST for computers to assess performance that included TR, TC, TE, and PERP.

The WCST was administered using the Psychology Experiment Building Language software package (the PEBL Project; http://pebl.sourceforge.net/). The WCST consists of four reference cards (left-to-right order: one red triangle, two green stars, three yellow crosses, and four blue circles) and 128 response cards [varying in shape (crosses, circles, triangles, or stars), color (red, blue, yellow, or green) and number (one, two, three, or four)]. The subject can be categorized according to color, shape, and number. The subject is subsequently requested to match each of the response cards to one of the four stimulus cards, but the criteria that must be followed to match the cards during the task are not indicated. After the response has been obtained, the letter on the screen indicates whether the letter has been correctly or incorrectly matched, but still does not disclose the correct classification principle. Thus, this forces the subject to repeat giving responses until the classification rule is discovered. Once the subject has correctly classified ten consecutive cards, the classification principles change without prior notice, so that the subject must modify his classification criteria to identify the new one. Overall, 128 WCST trials with no time limit were administered.

### Statistical analysis

Statistical analyses were performed in SPSS 26.0 (SPSS Inc). A one-way ANOVA and Fisher’s χ^2^ test were used to compare the continuous and categorical variables among groups, respectively. Two-way ANOVA with time (pre-TMS or post-TMS) as within-subject factors and TMS groups (HF-rTMS vs sham or iTBS vs sham) as between-subject factors was used to explore the effects of HF-rTMS and iTBS on working memory and executive functions, respectively. *Post hoc* analyses were performed using Sidak’s multiple comparison test. Cohen’s *d* and η^2^ was reported as a statistical effect. We set the significance level at *p* < 0.05.

To evaluate which was the most effective facilitatory protocol, a further *post hoc* analysis of normalized change from baseline was performed to compare the effects on cognitive function produced by the three excitatory protocols. The ANOVA or Kruskal–Wallis Test was used. The normalized change from baseline was defined as ([post-pre]/[maximum score-pre]); a positive value represents improvement while a negative value represents deterioration. Furthermore, the independent *t* test or Mann–Whitney was used to compare the effect sizes of iTBS and HF-rTMS on the improvement of working memory and executive functions (HF-rTMS vs sham, iTBS vs sham, and HF-rTMS vs iTBS), and the hypotheses were tested at the significance level of 0.0167 using two-tailed tests. Cohen’s *d* and η^2^ was reported as a statistical effect.

### Data availability

The data that support the findings of this study are available from the corresponding authors on request.

## Results

### Demographic and baseline data of the subjects

Out of the 71 participants who completed screening, 60 subjects were assigned to the HF-rTMS (*n* = 20), iTBS (*n* = 20), or sham (*n* = 20) groups (Fig. 1). Three subjects did not meet the inclusion criteria, 8 participants declined to participate. Neither the baseline demographics (sex, age, and level of education; Table 1), neuropsychological measures (MoCA, AVLT, SCWT, DST, CTT, VFT, HAMA, and HAMD; Table 1), nor the baseline scores of outcome measures (N-back, WCST; Table 2) showed any significant differences between the groups.

**Table 1:** Baseline characteristics of participants enrolled in the trial

Variable	Sham	20 Hz	iTBS	*F*/χ^2^	*p* value
	*N* = 20	*N* = 20	*N* = 20	df = 2,59	
Age (year)	23.50 (2.99)*	23.85 (2.79)*	23.80 (3.42)*	0.48*	0.785
Sex (male/female)	10/10	11/9	13/7	0.95^△^	0.622
Education duration (year)	17.40 (1.89)*	17.10 (1.69)*	18.00 (2.03)*	1.07*	0.587
MoCA	29.6 (0.38)*	29.45 (0.85)*	29.55 (0.47)*	0.22*	0.895
CAVLT-immediate	12.65 (1.18)	12.82 (0.98)	12.96 (0.93)	0.45	0.641
CAVLT-delay	14.75 (0.31)*	14.65 (0.33)*	14.75 (0.31)*	0.65*	0.721
CAVLT-recognition	14.85 (0.21)*	14.85 (0.21)*	14.85 (0.21)*	0.00*	1.000
HAMA	1.90 (1.80)	1.90 (1.62)	2.10 (1.65)	0.09	0.911
HAMD	1.30 (1.46)*	1.45 (1.28)	1.65 (1.58)*	0.62*	0.732
SCWT-dot (s)	12.31 (1.96)	12.40 (1.91)	12.37 (1.90)	0.01	0.988
SCWT-word (s)	14.76 (2.18)	14.84 (2.08)	14.77 (2.62)	0.01	0.993
SCWT-color word (s)	23.69 (5.43)	23.72 (5.13)	22.57 (4.48)	0.34	0.714
DST-Forward	9.45 (1.20)*	9.40 (1.20)*	9.65 (1.18)	0.59*	0.745
DST-Backward	7.15 (1.05)*	7.10 (1.08)*	7.30 (1.09)*	0.39*	0.821
CTT-A (s)	32.79 (9.31)	34.72 (11.14)	31.05 (9.09)	0.69	0.507
CTT-B (s)	65.98 (11.99)	65.75 (14.01)	66.49 (14.32)	0.02	0.984
VFT-sematic	20.13 (4.14)	20.10 (4.15)	20.85 (2.73)	0.26	0.772
VFT-Letter	9.50 (2.65)	9.00 (2.25)	9.15 (2.55)*	0.40*	0.819

Values in parentheses indicate SD; *, performed as median and interquartile range and Kruskal–Wallis Test; △, χ^2^ test.

MoCA, Montreal Cognitive Assessment; HAMA, Hamilton Anxiety Rating Scale; HAMD, Hamilton Depression Rating Scale; CAVLT, Chinese version of the Auditory Verbal Learning Test; DST, Digital Span Test; SCWT, Stroop Color Word Test; CTT, Color Trail Test; VFT, Verbal Fluency Test.

**Table 2:** Performance in N-back and Wisconsin card sorting task of participants at baseline

Variable	Sham	20 Hz	iTBS	*F*/χ^2^	*p* value
	*N* = 20	*N* = 20	*N* = 20	df = 2,59	
ACC-back 0 (%)	98.88 (1.63)*	98.88 (1.58)*	98.88 (1.68)*	0.03*	0.985
ACC-back 1 (%)	87.30 (8.44)	89.41 (7.47)	87.70 (9.07)*	0.56*	0.754
ACC-back 2 (%)	64.03 (17.97)	60.83 (18.49)	60.35 (17.98)	0.24	0.785
ACC-back 3 (%)	54.26 (24.23)	53.16 (21.78)	43.60 (20.22)	1.40	0.254
RT-back 0 (ms)	481.67 (65.26)	474.43 (51.76)	474.73 (52.38)	0.10	0.901
RT-back 1 (ms)	413.86 (133.64)	398.77 (131.1)	397.77 (112.21)	0.10	0.903
RT-back 2 (ms)	460.11 (187.06)	472.75 (185.08)	450.45 (162.66)	0.08	0.925
RT-back 3 (ms)	465.97 (150.40)	460.38 (180.65)	478.88 (179.08)	0.06	0.940
Total Correct	127.00 (17.00)*	128.00 (17.00)*	122.00 (21.50)*	1.75*	0.417
Total Correct	94.00 (9.75)*	94.00 (9.75)*	97.00 (9.75)*	0.07*	0.968
Total Errors	23.00 (22.00)*	24.00 (22.00)*	19.00 (19.50)*	1.68*	0.431
Perseverative errors percentage	10.63 (3.15)*	10.94 (2.95)*	9.02 (2.95)*	0.99*	0.611

Values in parentheses indicate SD; *, performed as median and interquartile range and Kruskal–Wallis test; △, χ^2^ test. ACC: Accuracy; RT: Reaction time.

### Effects of HF-rTMS for N-back and WCST

**Figure 2.**
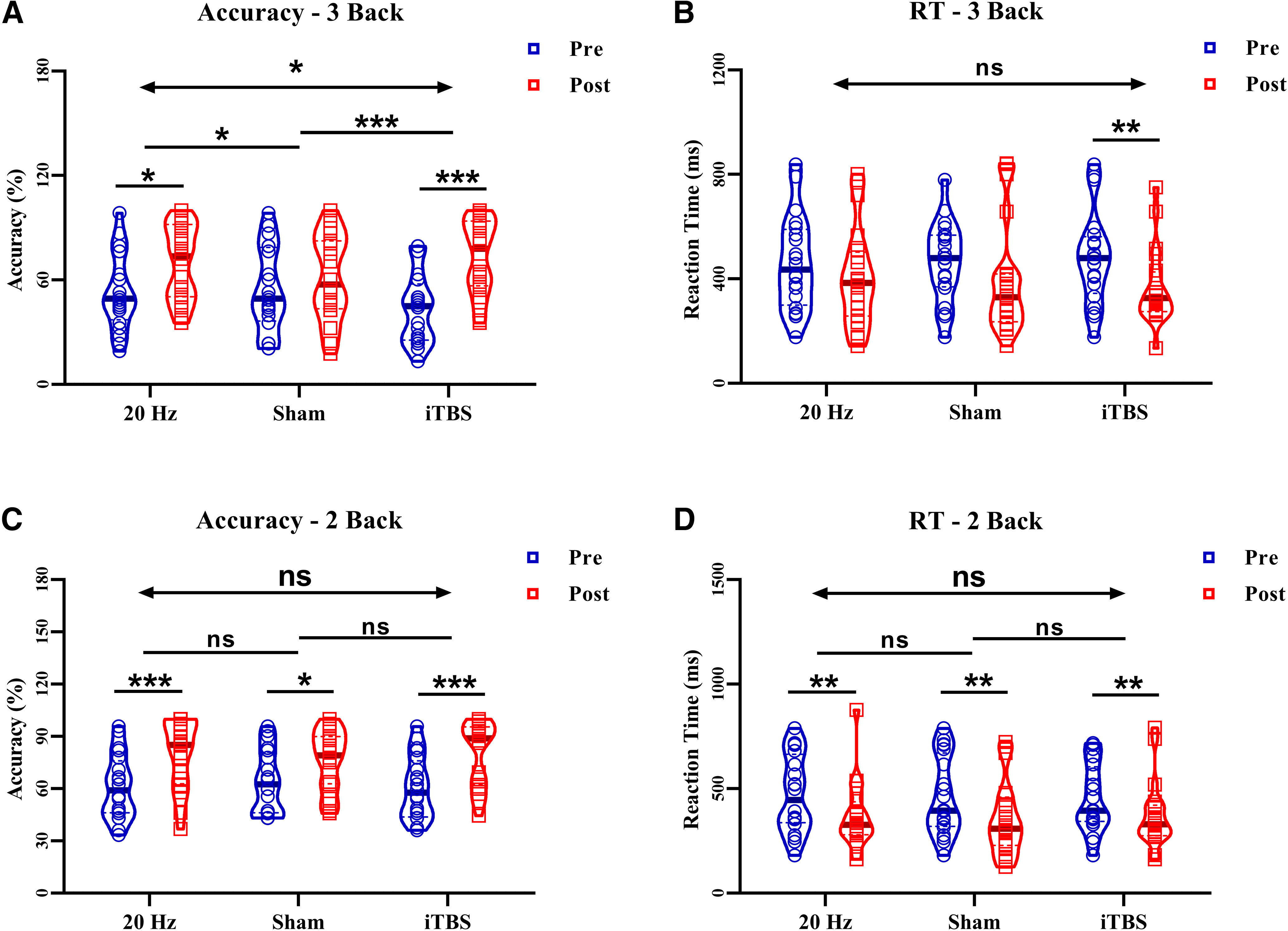
Score changes in the 2-back, 3-back for HF-rTMS, iTBS, and sham. ***A***, The ACC of 3-back shows a significant interaction effect after HF-rTMS, iTBS, and sham stimulation. The HF-rTMS and iTBS group shows a significant improvement after stimulation, but not in the sham group. Compare with sham group the enhancement of iTBS and HF-rTMS was significant. ***B***, The RT of 3-back shows no interaction effect after HF-rTMS, iTBS, and sham stimulation. The iTBS group shows a significant improvement after stimulation, but not the HF-rTMS. Compare with sham group the enhancement of iTBS was not significant. ***C***, The ACC of 2-back shows no interaction effect after HF-rTMS, iTBS, and sham stimulation. Both the HF-rTMS and iTBS and sham groups show an improvement after stimulation, but compared with the sham group, the enhancement of iTBS and HF-rTMS was not significant. ***D***, The RT of 2-back shows no interaction effect after HF-rTMS, iTBS, and sham stimulation. Both the HF-rTMS and iTBS and sham groups show an improvement after stimulation, but compared with the sham group, the enhancement of iTBS and HF-rTMS was not significant; ACC: Accuracy; RT: Reaction time; *p < 0.05, **p < 0.01, ***p < 0.001; ns: *p* > 0.05.

**Figure 3.**
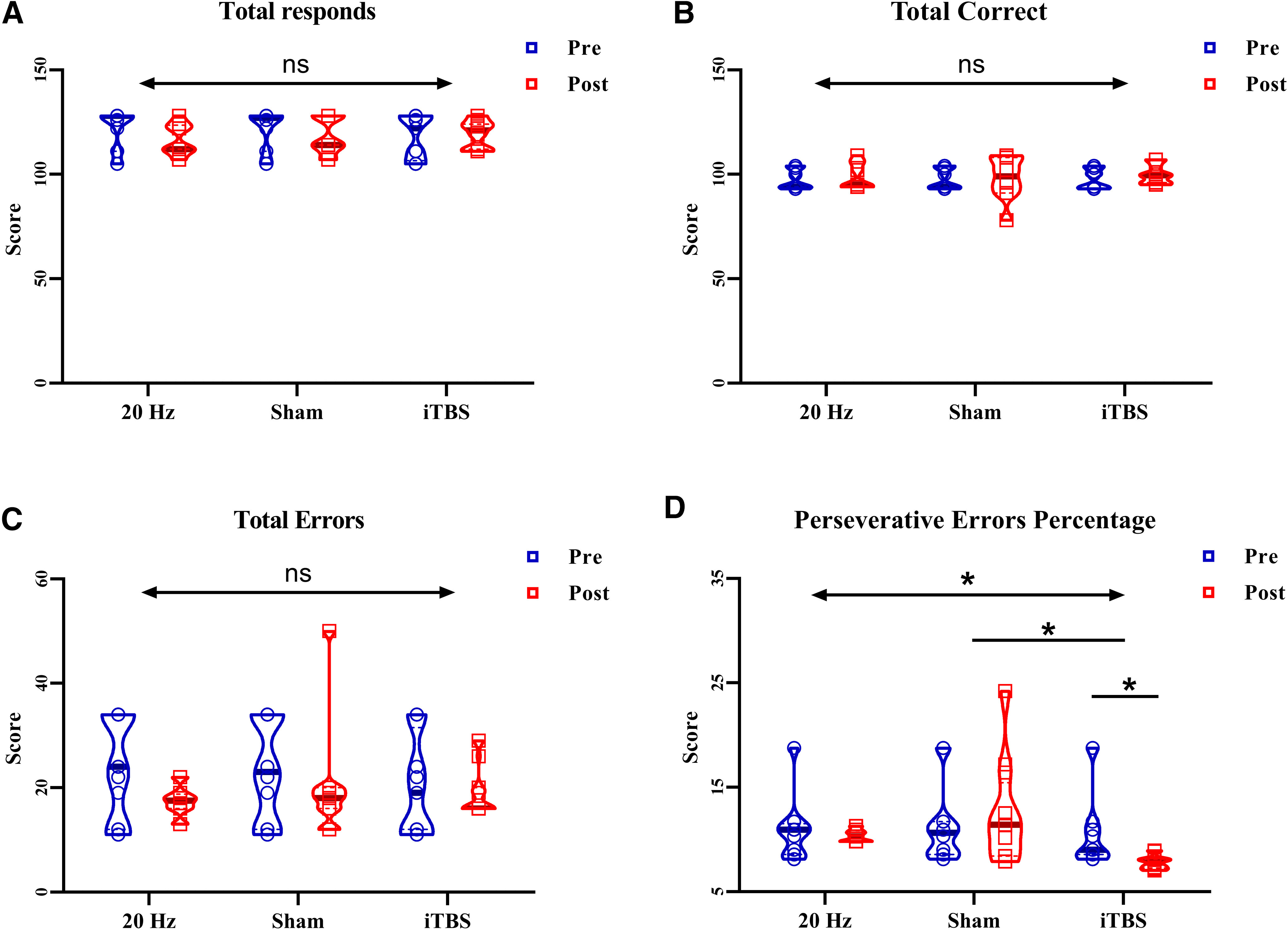
Score changes in WCST for HF-rTMS, iTBS, and sham. ***A–C***, The TR, TC, and TE of WCST shows no significant interaction effect after HF-rTMS, iTBS, and sham stimulation. ***D***, The perseverative errors percentage of WCST shows a significant interaction effect after HF-rTMS, iTBS, and sham stimulation. The iTBS group shows a significant improvement after stimulation, but not the HF-rTMS. Compared with the sham group, the enhancement of iTBS was significant, but not the HF-rTMS; *p < 0.05, **p < 0.01, ***p < 0.001; ns: *p* > 0.05.

In the ACC of the 3-back task, there was a significant interaction effect for time (pre vs post) and group (sham vs HF-rTMS; *F* = 4.92, *p *=* *0.033, η^2^ = 0.129; Fig. 2*A*,*B*). The ACC of the 3-back task was significantly improved in the HF-rTMS group (*t *=* *5.27, *p < *0.001; from 53.16% to 69.71%) relative to that in the sham group (*t *=* *2.13, *p *=* *0.078; from 54.27% to 60.96%), with an effect size of 0.71 (95% Confidence interval (CI) 0.06, 1.34) compared with the sham group. However, no significant interaction was observed in the accuracy of 2-back, 1-back, and 0-back tasks, RT of the N-back task (both *p *>* *0.05; Fig. 2*C*,*D*), and WCST (*p *>* *0.05; Fig. 3).

### Effects of iTBS for N-back and WCST

In the ACC of the 3-back task, there was a significant interaction for time (pre vs post) and group (sham vs iTBS; *F* = 16.67, *p *<* *0.001, η^2^ = 0.439; Fig. 2*A*,*B*). The ACC of the 3-back task was significantly improved in the iTBS group (*t *=* *7.46, *p < *0.001; from 43.60% to 73.16%) relative to that in the sham group (*t *=* *1.69, *p *=* *0.078; from 54.27% to 60.96%), with an effect size of 1.50 (95% CI 0.79,2.20) compared with the sham group. In the PERP of the WCST, there was a significant interaction for time (pre vs post) and group (sham vs iTBS; *F* = 7.09, *p *=* *0.011, η^2^ = 0.187; Fig. 3*D*). The PERP was significantly improved in the iTBS group (*t *=* *3.13, *p = *0.007; from 9.02% to 7.80%) relative to that in the sham group (*t *=* *0.63, *p *=* *0.779; from 10.63% to 10.78%), with an effect size of 0.84 (95% CI 0.19,1.48) compared with the sham group. However, no significant interaction was observed in the accuracy of 2-back, 1-back, and 0-back tasks; RT of the N-back task (both *p *>* *0.05; Fig. 2*C*,*D*), and TR,TC,TE of WCST (both *p *>* *0.05; Fig. 3*A*,*B*).

### Comparative effects of iTBS and HF-rTMS for N-back and WCST

**Figure 4.**
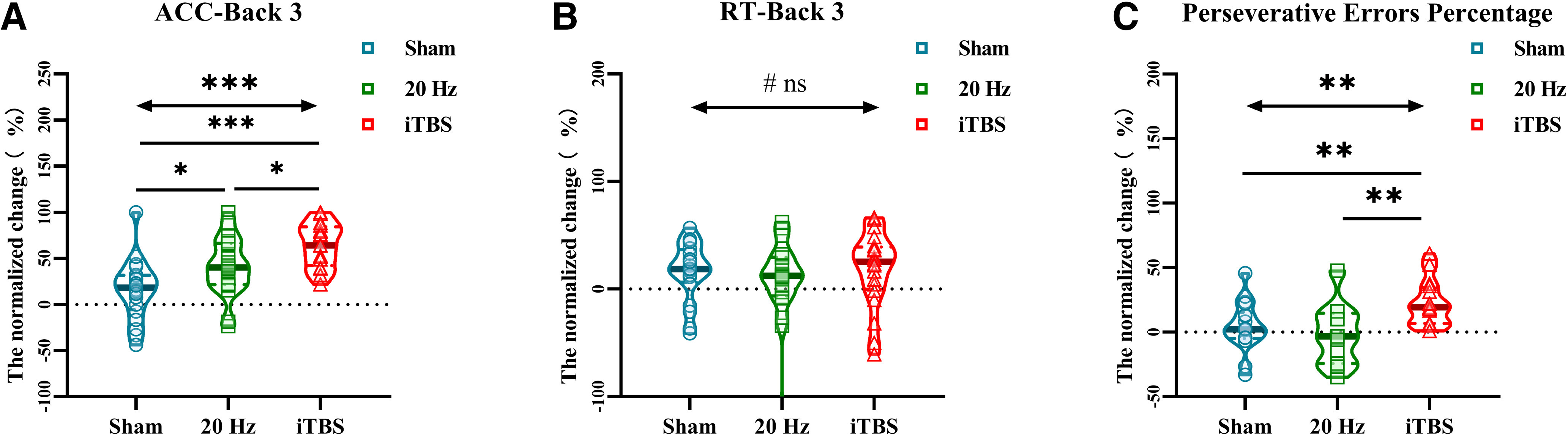
Comparison of normalized change from baseline in N-back and WCST after sham, HF-rTMS, and iTBS stimulation. (***A***) The improvement of 3-back accuracy by iTBS was significantly higher than that by HF-rTMS and sham. (***B***) There was no difference in the effects of iTBS, HF-rTMS, and sham on 3-back RT. (***C***) The improvement of perseverative errors percentage by iTBS was significantly higher than that by HF-rTMS and sham, but there was no difference in HF-rTMS and sham; *p < 0.05.

To compare the effect sizes between HF-rTMS and iTBS, the normalized change from baseline was analyzed. The results revealed that the promotion effects in the ACC of the 3-back task (*F*_(2,57)_ = 8.22, *p *=* *0.016, η^2^ = 0.012), the ACC of the 3-back task (*H*_(2,57)_ = 13.22, *p *<* *0.001, η^2^ = 0.017) and PERP (*H*_(2,57)_ = 10.37, *p *=* *0.006, η^2^ = 0.162) induced by different stimulus patterns are different. The iTBS showed higher effect sizes than 20-Hz rTMS in the ACC of the 3-back task (*t *=* *2.67, *p *=* *0.011; Fig. 4*A*,*B*) with a mean difference of 23.54% (effect size: 0.85; 95% CI 0.23,1.48) and PERP (*t *=* *3.57, *p *<* *0.001; Fig. 4*C*) with a mean difference of 23.34% (effect size: 1.13; 95% CI 0.48,1.79). But the difference was not observed in the accuracy of 2-back, 1-back, and 0-back tasks; RT of the N-back task; and TR,TC,TE of WCST (both *p *>* *0.05).

### Adverse effects

All participants tolerated iTBS, HF-TMS, and sham well, with no adverse events.

## Discussion

This study presents a randomized, double-blinded, sham-controlled design comparing the effects of sham stimulation, 20-Hz rTMS and iTBS, applied over the DLPFC, on working memory and executive functions in HC. First, a significant and large improvement in working memory performance was observed following active 20-Hz rTMS and iTBS. Second, the improvement of executive function (cognitive flexibility) performance was observed following iTBS, but not in 20-Hz rTMS. More importantly, we demonstrated that iTBS has a stronger promoting effect on the ACC of 3-back and PERP than 20-Hz rTMS. In summary, this study provides direct evidence that iTBS may be a better protocol for cognitive promotion.

Excitatory rTMS stimulation of left DLPFC can improve the working memory and cognitive flexibility in healthy adults, which is consistent with previous research (Bagherzadeh et al., 2016; Hoy et al., 2016; Chung et al., 2018). Cognitive flexibility is defined based on feedback from the experimenter; the participant can modify their behavior by identifying the appropriate strategy, which is an important part of executive function (Diamond, 2013; Guarino et al., 2019; Iimori et al., 2019). The DLPFC plays an important role in working memory and executive functions by encoding, extraction, strategy formulation, and cognitive flexibility (Barbey et al., 2013). Additionally, studies using various populations indicate that abnormal function of the DLPFC leads to abnormalities in working memory and executive function ability (Barbey et al., 2013; Yuan and Raz, 2014; Senkowski and Gallinat, 2015; Kumar et al., 2017; Naya et al., 2017). Studies of patients with mild cognitive impairment have reported that decreased excitability and impaired plasticity of the left DLPFC are connected to impaired working memory and cognitive flexibility (Yuan and Raz, 2014; Kumar et al., 2017; Guarino et al., 2018), while another study of patients with schizophrenia demonstrated that abnormalities in DLPFC oscillations are closely related to impaired working memory (Senkowski and Gallinat, 2015). A number of TMS studies have also demonstrated that disruptions in DLPFC function significantly reduce the performance of working memory and executive functions (Linden, 2007; Manenti et al., 2013; Burke et al., 2019). The effects and aftereffects of transcranial magnetic stimulation depend on the frequency and dose of rTMS. Different stimulation frequencies and pulses may induce different effects on the stimulation target and distal brain regions related to the stimulation target (Burke et al., 2019). Excitatory rTMS stimulation could improve blood perfusion, excitability, and plasticity of the target cortical region (Su et al., 2017). Generally, the higher the frequency, the stronger the excitability induced in the cortical areas, except with continuous TBS, which may have inhibitory effects (Hallett, 2007). Therefore, we speculate that the observed improvement in working memory and executive function performance may be related to changes in the excitability of the left DLPFC induced by TMS.

Improvements in cognitive function using iTBS compared with those using the traditional 20-Hz rTMS may be because of endogenous oscillations. It is important to note that the theta and γ bands have been linked to working memory processes (von Stein and Sarnthein, 2000; Vanrullen and Dubois, 2011; Hanslmayr et al., 2019). Theta oscillations play a critical role in the integration of the different brain regions required for working memory, with synchronous theta activity between prefrontal and posterior parietal regions associated with successful encoding in working memory (Sederberg et al., 2003; Sauseng et al., 2010). Furthermore, locally driven γ activity is thought to relate to information binding, reflecting ongoing neural computation, whereby increases in γ activity are thought to reflect enhanced sensory encoding processing, such as in N-back tasks (Sarnthein et al., 1998). The functional connection between the hippocampus and DLPFC may be achieved through the characteristic theta-γ phase-amplitude coupling, that is, the theta oscillation is superimposed on the γ oscillation, and the instantaneous γ amplitude waxes and wanes as a function of theta phase (Belluscio et al., 2012; Scheffer-Teixeira and Tort, 2016). The theta-γ coupling mechanism plays an important role in learning and memory as well as other cognitive functions (Colgin, 2015; Tamura et al., 2017). Transcranial magnetic stimulation has been shown to entrain cortical oscillations at its stimulation frequency (Thut et al., 2011b) and iTBS stimulation provides γ frequency stimulation (50 Hz) at theta frequency intervals (Huang et al., 2005), both of which have been strongly implicated in working memory functioning (Lisman, 2010). Hence, the enhancement of cortical activity specific to working memory induced by iTBS may explain the current findings in which iTBS produced notably large effects on working memory performance.

The iTBS may result in modulation of the excitatory (E)/inhibitory (I) balance to a more optimal ratio and thus primes the system for greater task-related neuronal synchrony and better cognitive performance (Hoy et al., 2016). Cortical activity is dependent on the E/I balance of synaptic inputs and there appears to be an optimal E/I ratio for maximal information capacity and information transmission (Beggs and Plenz, 2003; Shew et al., 2011; Yizhar et al., 2011). It has been theorized that noninvasive brain stimulation techniques induce improvements via modulation of this E/I balance (Krause et al., 2013). Intermittent TBS more closely mimics the brain’s natural neuronal firing patterns and produces more robust changes in cortical excitability, and subsequently enhanced neural firing and synchrony (Huang et al., 2005; Benali et al., 2011). In other words, iTBS may provide a more optimized cortical environment than the conventional rTMS protocol for improved performance of the task. However, further research including neural mechanisms is needed to prove this hypothesis.

The promotion of cognitive function by TMS may be limited, and different observation indicators need to be selected for different populations. The WCST was adopted to evaluate the role of the PFC in cognitive flexibility, especially the ability to switch between different task settings. Research based on neuroimaging has found that the left DLPFC plays an important role in the WCST, especially related to cognitive flexibility (Gläscher et al., 2019). In the current study, the 20-Hz rTMS protocol did not significantly improve WCST after stimulating the left DLPFC, which is inconsistent with previous research on psychiatric patients (Noda et al., 2018). Meaningfully, iTBS on the left DLPFC displayed a moderate effect size on the PERP which reflects the frontal cortex function of the subjects; however, the improvement of TR, TC response and TE response was not significant. This may be because of a ceiling effect, in that the subject’s WCST performance at baseline was close to the full score. In other words, there may be a certain upper limit on cognitive function promotion ability of TMS and the PERP may be a sensitivity indicator of executive function (Hoy et al., 2013).

There are several shortcomings in this experiment. First, only one unaccelerated HF-rTMS (20 Hz) model was adopted in this study. Future research should explore the effect of different frequency rTMS models (such as 5 Hz, 10 Hz, etc.), which mimic different endogenous rhythms on cognition. Second, the current study failed to explore the cumulative effects of TMS. In the future, the differences of cumulative effects between iTBS and different frequency rTMS should be assessed. Finally, this study did not explore the underlying neural mechanisms, and TMS-electroencephalogram and functional magnetic resonance imaging should be adopted to explore the details of neurologic mechanisms in the future. It should be noted that the sham stimulus in healthy subjects also has an improvement effect in N-back tasks, which may be influenced by a learning effect, in accordance with the results of other studies (Jones and Wilson, 2005; Tamura et al., 2017). Although a learning effect existed in all three groups, the sham-controlled design was adopted in our study to reduce the influence of learning effect as much as possible and further increase the reliability of the results by comparing the percentage of standardized changes in different groups.

In conclusion, the current findings are promising in that they reveal a more substantial effect of iTBS compared with the best conventional protocol of rTMS investigated to date. These findings indicate that iTBS may be a more appropriate and accelerated protocol for cognitive promotion in patients diagnosed with cognitive deficits related to diseases, such as Alzheimer’s disease and vascular cognitive impairment. However, further studies based on patients with cognition deficits are still needed to explore the effects of iTBS on cognition in real-world scenarios.

## Synthesis

Reviewing Editor: Karen Szumlinski, University of California at Santa Barbara Decisions are customarily a result of the Reviewing Editor and the peer reviewers coming together and discussing their recommendations until a consensus is reached. When revisions are invited, a fact-based synthesis statement explaining their decision and outlining what is needed to prepare a revision will be listed below. The following reviewer(s) agreed to reveal their identity: J. Nicole Bentley. Note: If this manuscript was transferred from JNeurosci and a decision was made to accept the manuscript without peer review, a brief statement to this effect will instead be what is listed below.

Upon consultation with 2 experts reviewers, we’ve have reached the decision of Revise and Re-view as the issues raised by the reviewers should require less than 2 months’ work to complete. Both reviewers considered the report worthy of publication in eNeuro pending the address of some major, but mostly minor comments, the majority of which relate to clarification of the text. While the comments are relatively minor, they are numerous as indicated below. Given the circumstances surrounding Covid-19, I completely understand if you require more than 2 months to complete the revisions and I ask that if you require extra time, to please contact the journal office and request an extension.

Reviewer 1:

This is a very interesting preliminary study examining whether one session of iTBS or 20Hz rTMS to L DLPFC may improve working memory and set-shifting performance compared to sham stimulation in a non-clinical group of young adults and whether change is more pronounced in iTBS over rTMS.

It is a well-planned experiment and the article will definitely be of interest to the readers. There are certain minor comments which need to be addressed:

1.Details of randomization approach need to be provided. How were participants allocated to groups?

2.”The coil was maintained horizontally pointing leftward, with its center positioned over the left DLPFC proper to maximize the strength of the electric field perpendicular to the target area.” Is the statement describing the coil positioning with the handle pointing in the posterolateral direction? It could be re-worded to describe the same.

3.A large number of tests were run. Was a multiple comparison correction applied? Although effect sizes large, would any of the identified results remain following correction for 16 tests completed in the main models using repeated measures ANOVA conducted and additional 12 tests looking at change scores?

Minor

1. Adverse effects were reported in results but not the method of recording adverse effects in methods

2. Were all the participants naïve to previous TMS experience?

Reviewer 2

Overall, this manuscript provides results of a sham-controlled trial comparing 3 different types of TMS. I think it is a good description of task results in a healthy population, however there are many areas that should be improved or addressed prior to considering publication.

Major concerns:

Significance Statement

L. 25: indicate whether double-blind or otherwise

Introduction

L. 56-58: What exactly is being “enhanced?” Is it an increase in power? Synchrony? If synchrony, what structures/frequencies are synchronized?

L. 59: What specific cognitive functions are improved? “Cognitive functions” is a very broad and vague term. In general, more specific detail is needed regarding which type of function.

L. 62: Please define what the standard approach is here.

L. 78: Please define in what structures this coupling occurs - is it within DLPFC itself, or between DLPFC and another structure. Is it phase-amplitude coupling, phase-phase coupling, or something else?

L. 82: Use of the word “enhancement” is vague. Do the authors mean improved accuracy, decreased reaction time, or something else?

Materials and Methods

L. 86: All 71 were randomized, but the control group would just constitute the sham stimulation group. Need to remove the word “control.”

L. 93: Would recommend changing this sentence to “sham-controlled, double-blinded (if true).”

L. 99: What was the rationale for why the tasks were performed after stimulation instead of during? The authors state the stimulation blocks were pseudorandomized - what was the period of time between stimulation sessions, and how did they account for possible washout effects (one stimulation protocol affecting the subsequent one) in their statistical analyses?

L. 124: Over what time was the iTBS given? Was it the standard protocol of 2 sec of stimulation followed by an 8-sec pause, repeated over 2 minutes?

L. 124: What is meant by “cumulative aftereffect” and why is this important?

L. 151: Was the N-back not also performed at baseline? There was no mention of this in section 2.5.1.

L. 158: The end of the sentence is missing.

L. 163: How is feedback given?

- How are these tasks administered (what kind of platform)? The WCST also incorporates changing rules - this is not mentioned. How many trials in the WCST?

L. 177-178: The sentence beginning “All the hypotheses” is redundant, stated above.

L. 181: How many comparisons does this significance level correct for?

Results

L. 186: Well-balanced is vague. Would recommend presenting this as whether nor not there were any significant differences in baseline demographics (list variables), neuropsych scores (list variables), or primary outcome measures (list variables).

L. 194: Please specify which “other tasks.”

L. 219: iTBS is mentioned, but what about HF-TMS and sham? Please detail the specific types of adverse events queried.

Discussion:

L. 224: The figures also show improvement in 2-back accuracy and reaction time before and after sham. This needs to be addressed in the discussion. Why would sham result in improvement? Could there be learning effects? How were possible learning effects addressed in the other stimulation conditions?

L. 226: The phrase “stronger cognitive promoting effect” is vague - recommend stating which effects specifically are improved (accuracy, RT?)

L. 233: I would be careful about designating behavior modification as the “most important” aspect of executive function. This is highly debatable.

L. 240: Again, “executive functions” is a vague and broad term. Which executive functions?

L. 246: What is meant by “regulation?"

L. 250: Some mention should be made of continuous theta-burst stimulation, where the same frequency, administered continuously, results in inhibition.

L. 264: “performed simultaneously” is confusing - it is not that the 2 oscillations occur at the same time, but that the peaks and troughs of one frequency (ie, the phase; usually in theta ranges) correlate to the phase or amplitude of another frequency band (here, gamma). Recommend clarifying this point.

L. 273-285: The definition of “priming", as presented in L. 284, should be moved ahead of the first sentence in the paragraph. The phrase “primed the cognition enhancement” is confusing as written.

L. 281: The definition of synchrony should be moved to the first mention of it, in the Introduction.

L. 301: Please clarify what is meant by “traditional rTMS model"

L. 304: What is meant by “cumulative effects?"

Conclusions

L. 310: I would not use the word “prove” to state that iTBS is the best protocol to use in disease states when the present study was limited to healthy controls. Healthy and diseased populations could be entirely different.

Tables and Figures

Table 1: The word “trail” should be “trial” in the table title.

The table overall is too long. Much of these scores could be included as a supplemental table. Alternatively, I would recommend breaking it into 2 smaller tables: demographics and neuropsychiatric assessments, and another table for experimental behavioral tasks. Consider presenting changes from baseline with each of the 3 conditions instead of raw scores, and presenting p-values.

Figure 1: The figure legend needs more of a description.

Figures 2-3: It seems that the more logical way of presenting this data would be to break this figure into 2, 1 for N-back and 1 for WCST. Then include the relevant significant tasks (3-back accuracy, RT, PERP) with all 3 stimulation conditions. This would allow easily comparing across the 3 conditions for improvement. The remaining tasks could be included in supplemental figures. Overall, because there are so many panels, the data points themselves are hard to see and the text is too small.

Also, the significant increase in accuracy, decrease in RT after sham stimulation needs to be addressed in the results/discussion.

Figure 4: Again, in order to improve readability, I recommend moving the non-significant findings to a supplemental figure and enlarging the text size/axes.

Minor concerns

- Throughout, the authors add a space between text and citations and no space. Recommend consistency throughout, with the space added.

- Recommend consistency in capitalizing “N” in “N-back"

Introduction

L. 35: “Parkinson’s disease, major depressive disorder (MDD), and schizophrenia.” Don’t need to capitalize.

L. 42: “Over the last several decades, ...”

L. 45: Would recommend commenting on the possible mechanism for this here.

L. 48: Recommend changing “functions” to “control"

L. 52: The first sentence is poorly written - the fundamental basis for all brain functions, whether pathologic, normal, or “improved” is likely related to oscillations, not just “cognitive function improvement” as the authors state.

L. 61: Don’t need to capitalize “Theta"

L. 61: Change “accelerate” to “accelerated"

L. 62: Need a comma after HF-rTMS

L. 62: Change to “...cortical excitability than the standard approach.”

L. 71: Need to define coherence

L. 78: Change “with” to “to"

L. 81: The acronym MDD has not been defined.

Materials and Methods

L. 88: State the criteria for exclusion, then report the number excluded and why

L. 98: Would change to “...different parameters was subsequently applied...”

L. 105: Change “visits” to “visit” and change to “evaluate global"

L. 123: Change “followed by a” to “followed at a"

L. 147: Change “indicating” to “indicate” and “stimulate” to “stimulus"

L. 147-148: Confusing as written - would reword to “...in the current trial, or 1, 2, or 3 steps earlier, responding by pressing a button.”

Discussion

L. 221: Would recommend re-stating as “double-blinded, sham-controlled” design

L. 222: Need to add the sham stimulation condition in addition to 20 Hz and iTBS

L. 230: Need comma after “controls"

L. 248: Recommend changing “related to” to “connected to"

L. 261: add “tasks” after “N-back"

L. 285: Need the citation for Hoy et al.

L. 285: Would change to “...may provide” - this point is not entirely known and shouldn’t be presented as conclusive.
